# Analysis of CT characteristics in the diagnosis of Schistosoma japonicum associated appendicitis with clinical and pathological correlation: a diagnostic accuracy study

**DOI:** 10.1007/s11604-019-00905-4

**Published:** 2019-12-10

**Authors:** Bimbadhar Valluru, Zhou Zhou, Dineswar Sah, Wei Du, Mahamed O. Ali, Ahmed A. Adam, Liang Zhang, Juan J. Wang

**Affiliations:** 1 The Department of Radiology and Interventional Surgery, The First Affiliated Hospital of Dali University, No- 32, Jiashi Bo Da Dao Road, Xiaguan, Dali, 671003 Yunnan People’s Republic of China; 2 The Department of Radiology and Interventional Surgery, Dali Bai Autonomous Prefecture Hospital, The Third Affiliated Hospital of Dali University, Dali, People’s Republic of China

**Keywords:** Schistosoma japonicum, Intestinal Schistosomiasis, Multislice computed tomography, curvilinear calcifications, Peri appendiceal abscess

## Abstract

**Purpose:**

To clarify unique non-contrast CT (NCCT) characteristics for early recognition of Schistosomal associated appendicitis (SAA) differentiating from Non-schistosomal associated appendicitis (NSA).

**Material and methods:**

Clinical and pathological data of 50 cases with SAA and 60 cases with NSA who underwent emergency appendectomy were retrospectively compared to pre-surgical NCCT features such as direct and indirect signs of acute appendicitis as well as appendicoliths, colon calcifications as diagnostic criteria. Statistical methods such as Chi-square (*χ*^2^), *t*-tests, Principal component analysis (PCA), Binary Logistic regression (LR) and Factor Analysis (FA) were utilized to observe differences and isolate recognizable CT features of SAA. Pre and post hoc diagnostic performance of all criteria was calculated as sensitivity, specificity, and the Odds Ratio (OR).

**Results:**

Age > 50 years, diameter > 13 mm, pneumatosis, peri appendiceal abscess, focal wall defect, perforation; Orbital, linear and point types of appendicular wall calcifications; sigmoid colon and cecal curvilinear calcifications were observed as unique characteristics with a sensitivity of 84–95% and specificity of 91–98% in predicting SAA by OR of 6.2 times. Pre and post hoc hypothetical analysis did not show any significance for all other factors.

**Conclusion:**

Factors such as elderly age, CT features such as larger appendicular diameter, appendicular wall calcifications along with sigmoid colon, and cecal calcifications, signs of perforation or abscess are characteristic for early recognition of SAA.

## Introduction

Schistosomiasis or bilharziasis is a chronic debilitating parasitic infection caused by Schistosoma japonicum, a trematode that is still highly prevalent as intestinal schistosomiasis in Asian countries [1]. It is also considered as the second most socioeconomically devastating condition after malaria by the World Health organization because of its high prevalence in Africa and the far east causing considerable mortality and morbidity [2, 3]. Especially in China, *S. japonicum* has reached a criterion of elimination, as well as, transmission interruption statuses with greater efforts [4], yet it remains a major health problem in the endemic villages of many provinces connected along Yellow river and Yangtze-Jiang basins [5, 6]. Despite the transmission control status, the focal transmission of *S. japonicum* is still vastly prevalent due to the risk of an epidemic rebound in autonomous regions of Yunnan Province especially like Dali, Lijiang, Chuxiong, and Honge city minorities. About 325,000 cases of hepatic and intestinal schistosomiasis were identified among a total population of 60 million in these regions, giving an incidence rate of 2.3% [7].

Schistosomal associated appendicitis (SAA) is a very special rare intitule causing acute appendicitis (AA) in 0.02–6.3% of patients prevailing from the non-endemic areas following a concomitant hematogenous ectopic localization of intestinal schistosomiasis due to the migration of *S. japonicum* eggs [8–10] into all parts of the gut including appendix eventually leading to abscess and granuloma formation [11, 12]. It presents with mild to severe clinical symptoms such as fever, abdominal pain, and vomiting or sometimes with unspecific symptoms such as unable to pass flatus, similarly as AA, the diagnosis is mostly dependent on clinical features in 70–80% of cases [13, 14]. But most patients become unsettling because it progresses very rapidly with spontaneous perforation, so it is often challenging to make a conclusive diagnosis just based on clinical and laboratory findings. However, a confirmatory diagnosis of SAA, as well as schistosomal granulomatous appendicitis (SGA), is often made by pathological studies, PCR- SCA ELISA and antischistosomal antibodies [15] following an emergency appendectomy. Given the possibility of SAA, pre-operative radiological investigations should be done with an aim to determine the etiological factor and severity assessment of illness is mandatory.

Based upon clinical symptoms and laboratory investigations, simple and equivocal appendicitis cases are often intervened with active clinical observation up to 24 h initially and primary antibiotic therapy respectively before or after establishing a diagnosis on preliminary radiological investigations like ultrasonography (USG), non-contrast CT (NCCT), contrast CT (CCT) [16]. Complicated appendicitis cases, on the other hand, receive broad-spectrum antibiotic therapy and radiological drainage. However, upon failure of initial management, operative appendectomy is the choice of management for all risk groups [17, 18]. Similarly, patients with SAA often undergo an emergency appendectomy, but delays in diagnosis and management are quite common when they present with unspecific symptoms. Praziquantel 20–60 mg/kg/dose divided into three doses for one day is recommended for all cases recognized as schistosomiasis and SAA after emergency surgery. Repeated doses after 2–6 weeks are usually required to improve the effectiveness of the therapy [19].

At present, several studies mainly focus on the clinical diagnosis, and management, etc., very few studies about radiological manifestations of SAA were carried all over the world as it is a neglected tropical disease (NTD). So, we conducted a retrospective study on 50 cases of schistosomal associated appendicitis and 60 patients of non-Schistosomal associated appendicitis to verify the characteristic radiological features and factors influencing the prediction of SAA outcome. We anticipate that our study could provide a detailed radiodiagnosis of SAA as well as, helping the diagnostic clinicians and radiologists for early recognition, risk estimation, isolation of these cases and carry effective interventions in the endemic regions of Schistosomiasis.

## Materials and methods

### Study population

Ethical approval for all the materials and methods was given by the institutional organization for clinical drug trials and scientific research division. All the protocols were carried out in accordance with the guidelines and regulations set by the department of radiology and interventional surgery. Informed written consent on standard forms from all the patients was obtained for interventions such as imaging series, surgical procedures, histopathology specimen collection, the use of clinical data and research.

Clinical and pathological data of 572 patients who presented to the emergency department with acute appendicitis between March 2015 and January 2019 were collected. Details such as age, sex and ethnic origin of all the patients were recorded. Appendicitis inflammatory response score (AIR) [17, 18], as well as the Alvarado score [20], were also calculated based on presenting symptoms and laboratory investigations simultaneously. The clinical risk of acute appendicitis was estimated and classified as necessary.

Based on these clinical scores, all the patients were forwarded to either presumed radiological or clinical, surgical interventions as necessary. 15 patients did not meet clinical criteria for AA and were managed with empirical treatment; 149 patients received clinical and/or surgical management directly with no prior radiological investigations; 78 patients received USG and 22 patients had post-surgical CT scanning only.

**Fig. 1:**
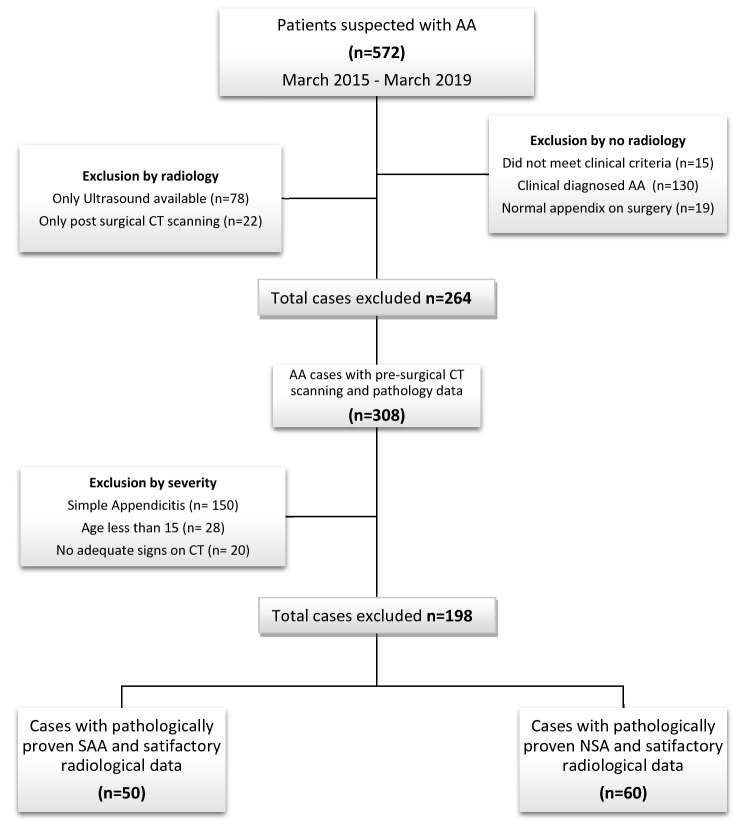
Flow chart outlining the study population. *AA *acute appendicitis, *SAA *schistosomal associated appendicitis group, *NSA *non-schistosomal associated appendicitis group

308 cases were recommended for the pre-surgical diagnostic estimation of AA severity by non-contrast CT (NCCT), out of which, 150 cases showed only appendiceal enlargement > 6–8 mm and inflammatory signs, so they were recommended with antibiotic treatment. All 308 cases underwent emergency appendectomy within 12–24 h of admission in the respective general surgery department following histopathological study. Only 110 cases with pre-surgical CT scanning demonstrating adequate signs of acute appendicitis with a determined etiological factor on pathology were included in this study. A total of 462 cases were excluded from the study as demonstrated (Fig. 1).

**Fig. 2:**
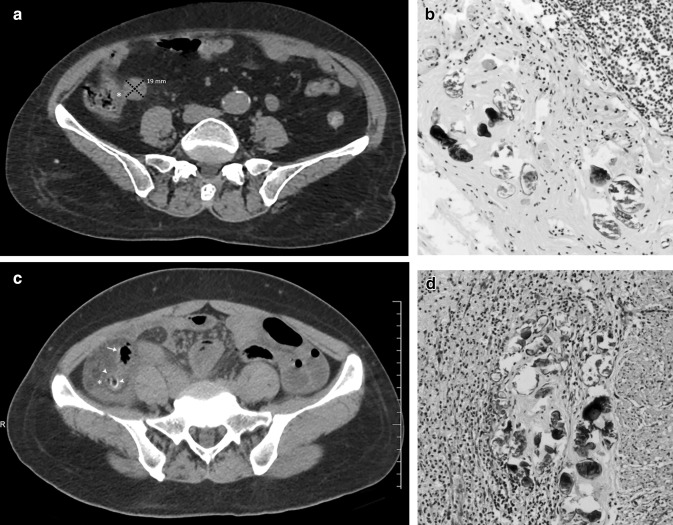
NCCT axial sections of SAA cases **a** A 69-year-old female with suppurative type SAA demonstrating inhomogeneous densities (asterisk) near enlarged appendix with a diameter of 19 mm (marked) suggesting abscess formation; **b** Postoperative pathological section of the same patient showing hyperemic and edematous appendix with diffuse inflammatory cell infiltration and large partially calcified eggs; **c** A 54-year-old female with granulomatous type SAA showing an appendicolith in the lumen (arrowheads) with the typical mural calcification along the wall of the appendix (arrow). Note heterogeneous densities and effusions around the appendix (asterisk); **d** Postoperative pathological section of the same patient showing hemorrhagic necrosis, inflammatory cell infiltration along with fibrosis, calcified egg deposition and granuloma formation in every layer (Hematoxylin and Eosin stain ×400)

Pathological specimens of all these cases showed varying degrees of acute and chronic inflammatory cells infiltration, congestion and swelling, suppuration, necrosis and perforation of the appendiceal wall collectively suggesting a diagnosis of acute suppurative (ASA) and acute granulomatous appendicitis (AGA). Apart from the findings of AGA and ASA, 50 cases also revealed *S. japonicum* eggs deposition, calcifications, and chronic granulomatous nodules on histopathology (Fig. 2b, d).

### Grouping

Finally, 50 cases with a confirmatory diagnosis of Schistosomal associated appendicitis (SAA) established from pathological examinations selected on convenience case series constituted the observational group—SAA group. These cases belong to the Dali Bai minority group from ZhiZhou county and surrounding schistosomiasis endemic regions of Yunnan Province with a definitive history of exposure to contaminated upstream waters for 28–65 years (mean 47) of a lifetime. 60 cases with a confirmatory diagnosis of non-schistosomal associated appendicitis (NSA) secondary to streptococcus, pseudomonas presenting as mucosal, ASA and AGA were selected to form the control group- NSA group.

### Radiological parameters

NCCT scanning was performed on *Phillips Brilliance* 16 slice CT scanner with scanning range from the diaphragm to symphysis pubis; scanning parameters standard was set to tube voltage 120kv; tube current 150–250mAs; pitch 0.938; X-ray tube rotation 0.5 *r/s*; matrix 512 × 512; conventional scanning: layer thickness 3 mm and increment 3 mm; for multiplanar reconstruction (MPR): layer thickness 2 mm and increment 1 mm.

### Comparative analysis of the image

In the current study, pre-surgical CT features of acute appendicitis were compared between the SAA and NSA groups (the high-risk group of appendicitis with suspected perforation and abscess formation) and analyzed with clinical and pathological data retrospectively.

Multi-slice compilations were transferred to advanced PACS workstation (*SEAN 2.0*) for a multiplanar reformation (MPR) visualizing complete abdomen with appendix, adjacent viscera including liver and colon in transverse, sagittal and coronal sections respectively. All the cases were independently analyzed by 3 abdominal radiologists and were blinded from confirmatory diagnosis. Radiographic features such as the diameter of appendix > 6–22 mm, the thickness of the appendicular wall > 3 mm, peri-appendiceal streak shadows or inflammation, effusions > 2.6 mm, fluid collections, right lower quadrant reflex bowel dilation (RLQRBD), peri appendicular abscess, pneumatosis, appendicolith(s) (CT value > 80 HU), and colon wall calcification(s) were considered as the diagnostic criteria. Abnormal findings of the abdominal viscera within the scanning range were also recorded. An independent abdominal radiologist provided a consensus to eliminate the evaluation bias. All images were observed at a low window level  − 35–35HU, and the window width of 300–400HU.

### Statistical analysis

Continuous variables in each group were expressed as the mean ± standard deviation (SD) for normally distributed data and categorical data were expressed as the number of patients and percentages. Based on the results of the homogeneity test of variance, group comparisons were made using a t-test for measurement data; count data were analyzed using the chi-square (*χ*^2^) test with continuous correction. *P* < 0.05 was considered significant. Pre-hypothetical diagnostic performance of all parameters was calculated individually as sensitivity, specificity, positive predictive value (PPV), negative predictive value (NPV) and accuracy in predicting SAA.

All the parameters were entered into Principal component analysis-PCA accordingly; with determinant value 0.001, Kaiser Meyer Olkin measure of sampling adequacy (KMO) (0.662), Bartlett’s Test of Sphericity (0.001), a reliable model of 4 components (C) {1:2:3:4} from 14 variables was extracted, which showed collinearity within interrelated variables (diagnostic criteria). Therefore, the respective factor regression scores were calculated. The differences between these scores were analyzed using binomial logistic regression (LR) obtaining Odds ratio (OR), 95% Confidence interval (CI).

Based on this data, Factor analysis (FA) was performed with Promax rotation excluding non-significant components on LR to obtain a hypothetical association and confirm the relations to SAA within the model-data structure of considered parameters; the strength of relationship among the variables extracted a conjugation of 3 factors within 8 interrelated variables. The percentages of influence on each factor were calculated on the respective rotated matrix finally obtaining significant radiographic features related to SAA. Score distributions (minimum-1 and maximum-3) to each isolated parameter was given accordingly; a composite severity score for prediction of SAA outcome was calculated. The post hypothetical diagnostic performance of these factors and were analyzed on binomial LR. The results were expressed as corresponding OR, 95% CI, the area under the curve (AUC), cutoff values, sensitivity, specificity.

Data management and complete statistical analysis were done with MedCalc 18.11, 2019 *(MedCalc Software, Ostend, Belgium)* and SPSS Statistics 25.0, 2019 *(IBM SPSS, Chicago IL)* software packages.

## Results

### Clinical data

Out of 50 cases of the SAA group, there were 26 men and 24 women; in 60 cases of NSA group, there were 35 men and 25 women (*p* = 0.318). The average age of onset of appendicitis in the SAA group was 62.82 ± 12.74 years with age > 50 years showing the highest positivity rate (86%); the average age of onset of appendicitis in NSA group was 41.58 ± 18.82 years (*p* = 0.014). AIR score for indeterminate risk (5–8) in 52.7% and high risk (9–12) in 47.3% of cases (*p* = 0.087); a probable diagnosis of AA was observed in more than 82.7% of cases for Alvarado score (*p* = 0.457) (Table 1).

**Table 1:** Clinical data

Clinical criteria	Number of cases with AA		Observation	*P*-value
Groups	SAA (*n* = 50)	NSA (*n* = 60)	*n* = 110	N/A
Age* (years)	62.82 ± 12.74	41.58 ± 18.82	N/A	0.014
Sex (M/F)	26/24	35/25	M: F = 1.2:1	0.318
*AIR score*			*Total*	
Indeterminate risk (I: 5–8)	31 (28.2%)	27 (24.5%)	52.7%	0.087
High risk (H: 9–12)	19 (17.3%)	33 (30.0%)	47.3%	
*Alvarado score*			*Total*	
Compatible – probable AA	43 (39.1%)	48 (43.6%)	82.7%	0.457
Very probable AA	7 (6.3%)	12 (11.0%)	17.3%	

*Mean age ± standard deviation (*SD*), *N/A *not applicable, *M *male/men, *F *Female/women,

*AIR *Appendicitis inflammatory response score, *AA *Acute appendicitis

### CT Imaging findings

According to the considered diagnostic criteria, CT revealed the following findings for the experimental and the control groups (SAA/NSA), respectively.

- All the cases showed enlarged appendix and varying degrees of appendiceal wall thickening (basic signs) therefore, they did not show significant statistical differences (*p* > 0.05) except for appendicular diameter (*p* = 0.007) vividly seen > 13.6 mm in most cases of SAA group (Fig. 2a).
- Direct and indirect signs of complicated appendicitis appear to have a higher incidence in the SAA group (Fig. 2a, b). They were either significant or not significant suggesting complicated appendicitis prompting the need for calculation of the percentage of influence individually for differentiation of SAA from NSA (Table 2).

**Table 2:** Pre- hypothetical diagnostic performance of CT diagnostic criteria

Observed features^Ϯ^	No of cases		Sensitivity (%)	Specificity (%)	PPV* (%)	NPV* (%)	Accuracy (%)	*P* value^#^
	SAA group (*n* = 50)	NSA group (*n* = 60)						
Diameter of the appendix (mm)	13.65 ± 2.14	11.05 ± 2.06	-	-	-	-	-	**0.007**
Peri-appendiceal inflammation or streak shadows	46 (92%)	59 (98%)	46/105 (44)	1/5 (20)	46/50 (92)	1/60 (2)	43	0.130
RLQ bowel dilatation	39 (78%)	46 (77%)	39/85 (46)	14/25 (56)	39/50 (78)	14/60 (23)	48	0.520
Appendicolith	26 (52%)	25 (42%)	26/51 (51)	35/59 (59)	26/50 (52)	35/6 (58)	56	0.187
Focal wall defect	13 (26%)	8 (13%)	13/21 (62)	52/89 (58)	13/50 (26)	52/60 (87)	59	0.092
Effusions	23 (46%)	18 (30%)	23/41 (56)	42/69 (61)	23/50 (46)	42/60 (70)	59	0.060
Peri appendiceal abscess	15 (30%)	5 (8%)	15/20 (75)	55/90 (61)	15/50 (30)	55/60 (92)	64	**0.003**
Pneumatosis	15 (30%)	8 (13%)	15/23 (65)	52/87 (60)	15/50 (30)	52/60 (87)	61	**0.028**
Perforation	11 (22%)	5 (8%)	11/16 (68)	55/94 (58)	11/50 (22)	55/60 (92)	60	**0.043**
*Appendiceal wall calcification*								
Point type calcification	7 (14%)	2 (3%)	7/9 (78)	58/101 (57)	7/50 (14)	58/60 (97)	59	**0.042**
Linear type calcification	26 (52%)	4 (7%)	26/30 (87)	56/80 (70)	26/50 (52)	56/60 (93)	75	** < 0.001**
Orbital type calcification	43 (86%)	1 (2%)	43/44 (98)	59/66 (89)	43/50 (86)	59/60 (98)	93	** < 0.001**
Colon calcifications	45 (90%)	7(12%)	45/52 (87)	53/58 (91)	45/50 (90)	53/60 (88)	89	** < 0.001**

^Ϯ^Complicated appendicitis-Direct signs: Diameter > 8 mm, wall thickening > 3 mm, effusions > 2.6 mm, fluid collections, intra/extraluminal pneumatosis, appendicolith, fat stranding; Indirect signs: RLQ bowel dilatation, peri appendiceal inflammation or streak shadows, focal wall defect, abscess formation, perforation (likely or impeding)

^*^PPV Positive predictive value, *NPV* negative predictive value

^#^Significant values marked in bold
- Pre-hypothetical diagnostic performance of CT signs did not yield satisfactory sensitivity and specificity standards for basic appendicitis criteria but yielded considerable levels for appendiceal wall calcifications and colon wall calcifications; however, for all the parameters that were significant on *χ*^2^ and *t*-tests- PPV, NPV and accuracy levels were not adequate to predict SAA outcome (Table 2).
- The presence of appendicoliths was observed in almost 40–50% of cases from both the groups demonstrating heterogeneous to homogeneous hyper densities within the appendicular lumen. There was no significant statistical difference (Figs. 2c, 3a–d and 4a).

**Fig. 3:**
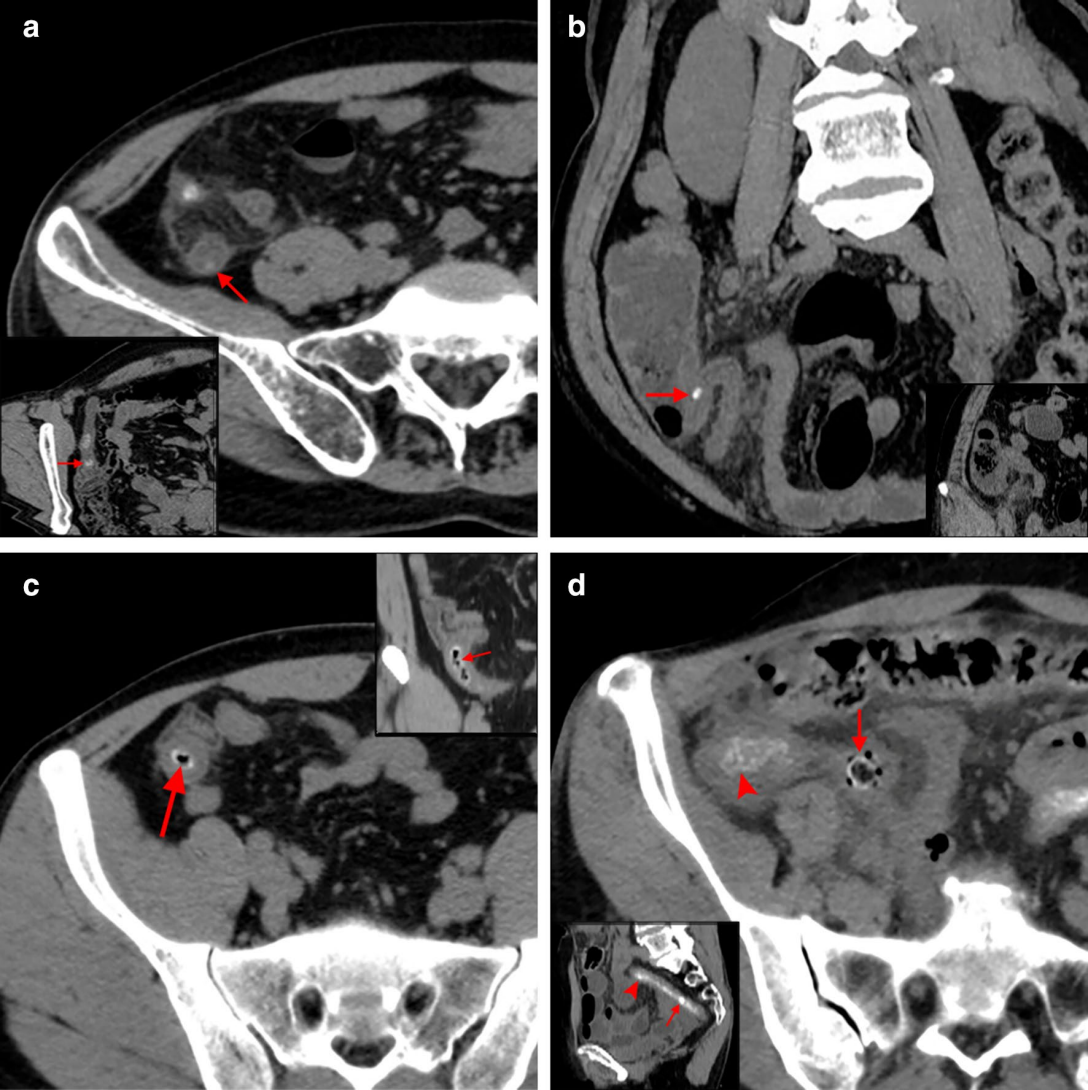
NCCT sections showing differentiation of appendicolith from wall calcifications **a** Axial view of a 42-year-old male with suppurative type NSA showing the presence of appendicolith (arrow), CPR with oblique rotation (coned out region) revealing the appendicolith inside the lumen (arrow); **b** Coronal view of a 57-year-old female with gangrenous perforated type NSA showing the presence of appendicolith obstructing the lumen of an enlarged appendix (arrow), MPR coronal view with anterior increment (coned out region) showing fat stranding and peri appendiceal streak shadows; **c** Axial view of 28- year-old with suppurative type SAA showing an orbital calcification (arrow) mimicking as appendicolith; sagittal view (coned out region) reveals hyperdense calcifications are indeed present along the inner wall of the appendix (arrow); **d** Axial view of 56- year- old female with gangrenous perforated type SAA showing appendicolith with surrounding pneumatosis (arrow) and appendicular wall mural calcifications (arrowhead); sagittal section (coned out region) showing clear demarcation of appendicolith inside the lumen and wall calcifications

**Fig. 4:**
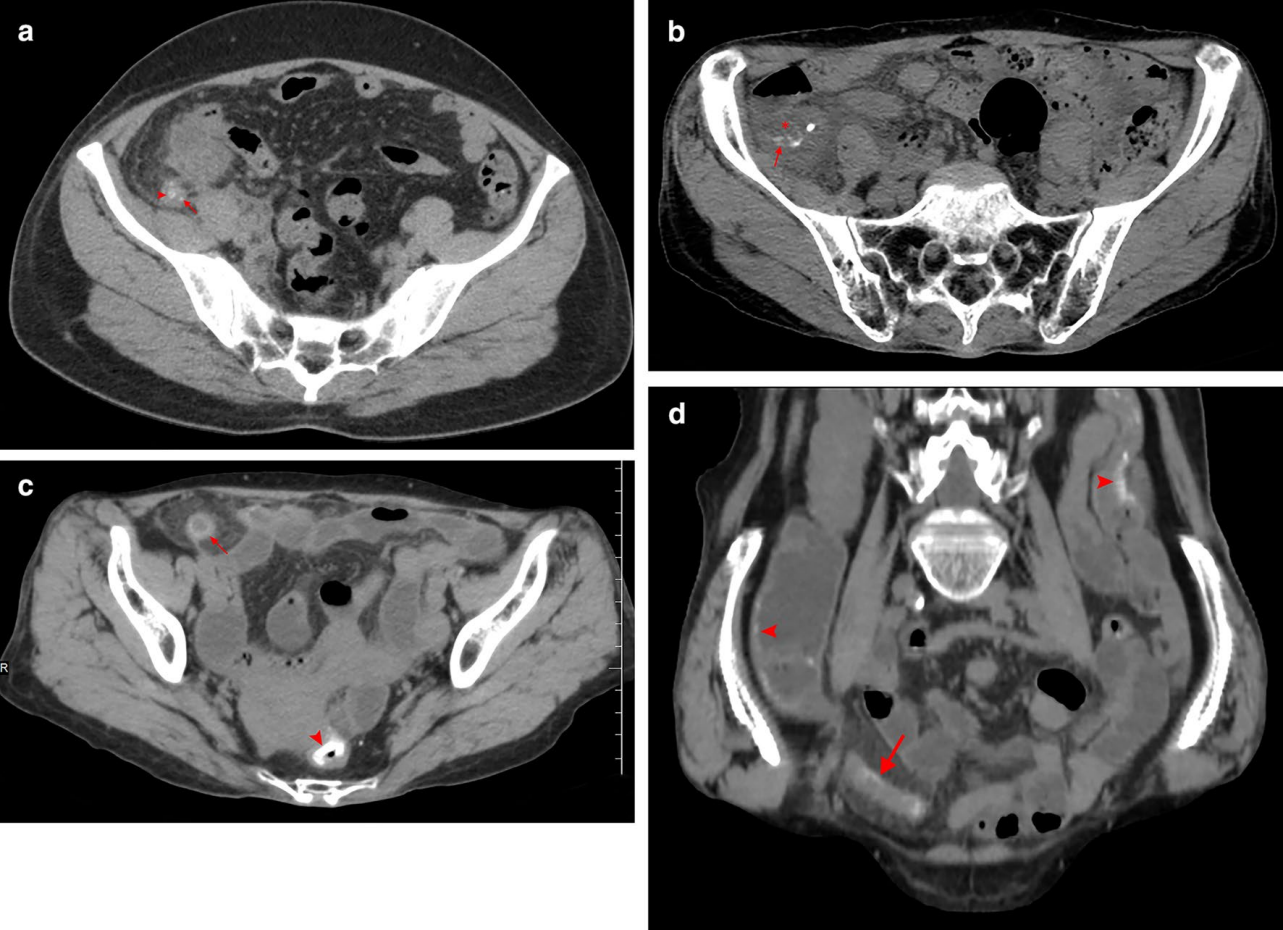
NCCT sections of SAA cases demonstrating typical appendicular calcifications **a** Axial view of 54-year-old female showing hyperdense point type calcifications (arrow) along the appendicular wall with an appendicolith in the lumen (arrowhead); **b** Axial view of 74-year-old female showing hyperdense linear type calcifications (arrow). Note the presence of point type calcification, heterogenous streak shadows, effusions around enlarged appendix (asterisk); **c** Axial view of 71-year-old female showing hyperdense orbital type annular calcification along with dilated appendix (arrow); **d** Coronal view with an oblique rotation of the same patient showing the extent of calcifications along the full length of the appendicular wall (arrow). Note the presence of colonic calcifications in both views (arrowheads)
- Appendiceal calcifications were also observed on MPR/CPR which revealed 3 sub-types: (1.) Point type, single to multiple focal hyper densities close to the inner wall or outer wall of the appendix (Fig. 4a) (2.) Linear type, a streak-like hyper densities extending along the length of appendix peripherally close to the inner wall in two planes (Fig. 4b) (3.) Orbital type, circular to oval mural hyper densities along the length and breadth of the appendix in all planes (Fig. 4c–d). These calcifications showed significant statistical differences in both groups respectively, out of which orbital calcification was a frequent finding in the SAA group (86%).
- SAA group demonstrated the presence of typical curvilinear type calcifications i.e. uniform and continuous hyperdensities along the colon wall in multiple locations involving rectum and sigmoid colon dominantly (Fig. 4c–d and Fig. 5a–c). However, striated and striped calcifications were also mostly seen in many cases along the cecum or ascending colon leading to the poor filling of the intestine (Fig. 4d and Fig. 5c). 15 cases in the SAA group also showed capsular calcifications along the liver parenchyma (Fig. 5d); Colon calcifications in NSA group are due to known secondary conditions.

**Fig. 5:**
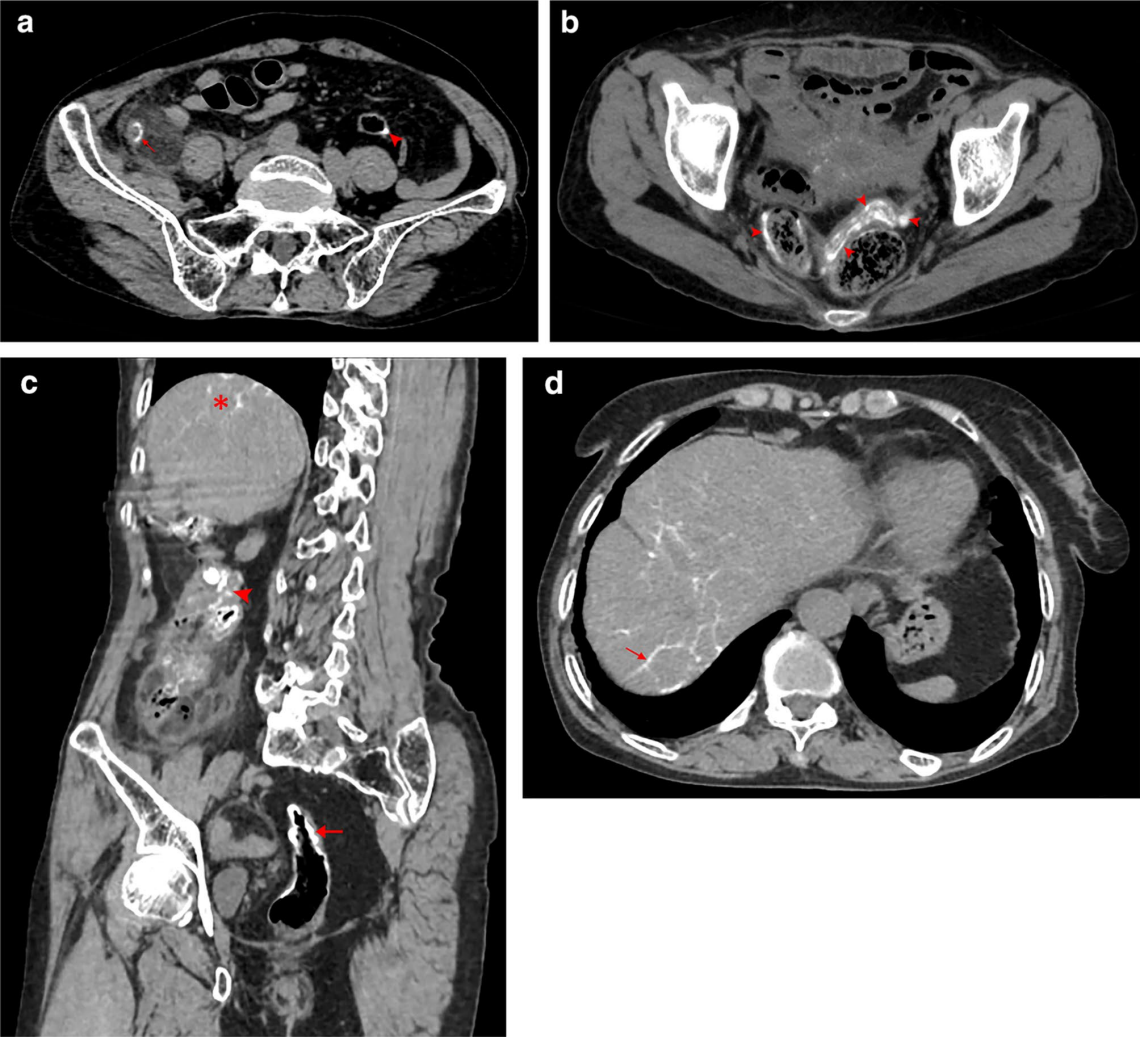
NCCT imaging of 84-year-old female with SAA **a** Axial view at appendicular level showing dilated appendix with orbital calcification (arrow) and descending colon calcification (arrowhead); **b** Axial view at sigmoid colon level demonstrating characteristic tram track or curvilinear calcifications (arrowheads); **c** Sagittal view with oblique rotation in MPR visualizing calcifications along caecum and ascending colon (arrowheads) as well as sigmoid colon (arrow). Note the liver calcifications (asterisk); **d** Axial view showing hyperdense characteristic “turtleback” capsular calcifications along liver parenchyma (arrow)

### Hypothetical analysis

Binary logistic regression model analysis of extracted regression scores of 4 components on PCA showed significant differences between C{1:2:3} except for C{4}. C1 has shown a probability of 2 times in determining the SAA outcome individually (Table 3). After Promax rotation with Kaiser normalization, the factors loadings on the structure matrix of FA were interpreted as follows (Table 4):

- Sigmoid colon calcifications with multiple locations (0.924), point type (0.893), linear type (0.697) and orbital type (0.940) calcifications, age > 50 years (0.758) and appendicular diameter > 15 mm (0.569) has the largest positive loadings on factor 1; suggesting all primary radiographic features necessary for differentiating SAA from NSA.
- Focal wall defect (0.957) is associated with the perforation (0.954) on factor 2; Pneumatosis (0.975) is associated with the presence of abscess (0.971) on factor 3, has the largest positive loadings respectively suggesting secondary features and associated complications for correlation.

**Table 3:** Principal component analysis and binomial logistic regression

Component^#^	Criteria*	B	Sig	Exp (B)	95% CI for Exp (B)	
					Lower	Upper
C1	Colon calcification; Orbital type; Linear type; point type; Age; Appendicular diameter; Abscess; Pneumatosis	0.745	0.001	2.106	1.465	3.029
C2	Abscess; Pneumatosis	− 0.977	0.001	0.376	0.217	0.653
C3	Perforation; focal wall defect	0.492	0.045	1.636	1.010	2.651
C4	RLQBD; peri appendiceal shadows; appendicolith; effusions	0.094	0.698	1.098	0.684	1.764
	Constant^Ϯ^	0.212	0.360	1.237		

^#^Variables extracted on C1–C4 = Component 1, Component 2, Component 3 and Component 4

^*^The Hosmer–Lemeshow test for goodness of fit shows that the chi-square value is 14.229 with *p* = 0.076; overall percentage = 54.5;

^Ϯ^Model summary:  − 2 log likelihood 113.387; Cox & Snell R square 0.293; Nagelkerke R square 0.392

**Table 4:** Factor analysis and SAA scoring

Criteria	Variable	Parameter*	Factor structure matrix^#^			Score distributions^Ϯ^
			Primary features (1)	Secondary features (2) complications (3)		
Colon wall calcification	X1	Any location except sigmoid colon	− 0.653			1
	X2	Only sigmoid colon	0.782			2
	X3	Sigmoid colon with multiple locations	0.924			3
Appendicular wall calcifications	X4	Point type	0.893			1
	X5	Linear type	0.697			1
	X6	Orbital type	0.940			2
Age	X7	> 50 years	0.758			1
Diameter	X8	10–15 mm	0.525			1
	X9	> 15 mm	0.569			2
Others	X10	Focal wall defect		0.957		1
	X11	Perforation		0.954		
	X12	Pneumatosis			0.975	1
	X13	Abscess			0.971	

^#^Extraction method: factor analysis; rotation method: Promax with Kaiser Normalization converged in 5 iterations; The determinant value on the correlation matrix is 0.02; the KMO measure of sampling adequacy is 0.678 and Bartlett’s Test of Sphericity is 0.001

^*^Coefficient values > 0.5 (50%) was set as the absolute cut-off value for sorting

^Ϯ^Score distributions were based on the percentage of influence on structure matrix and BLR

**Fig. 6:**
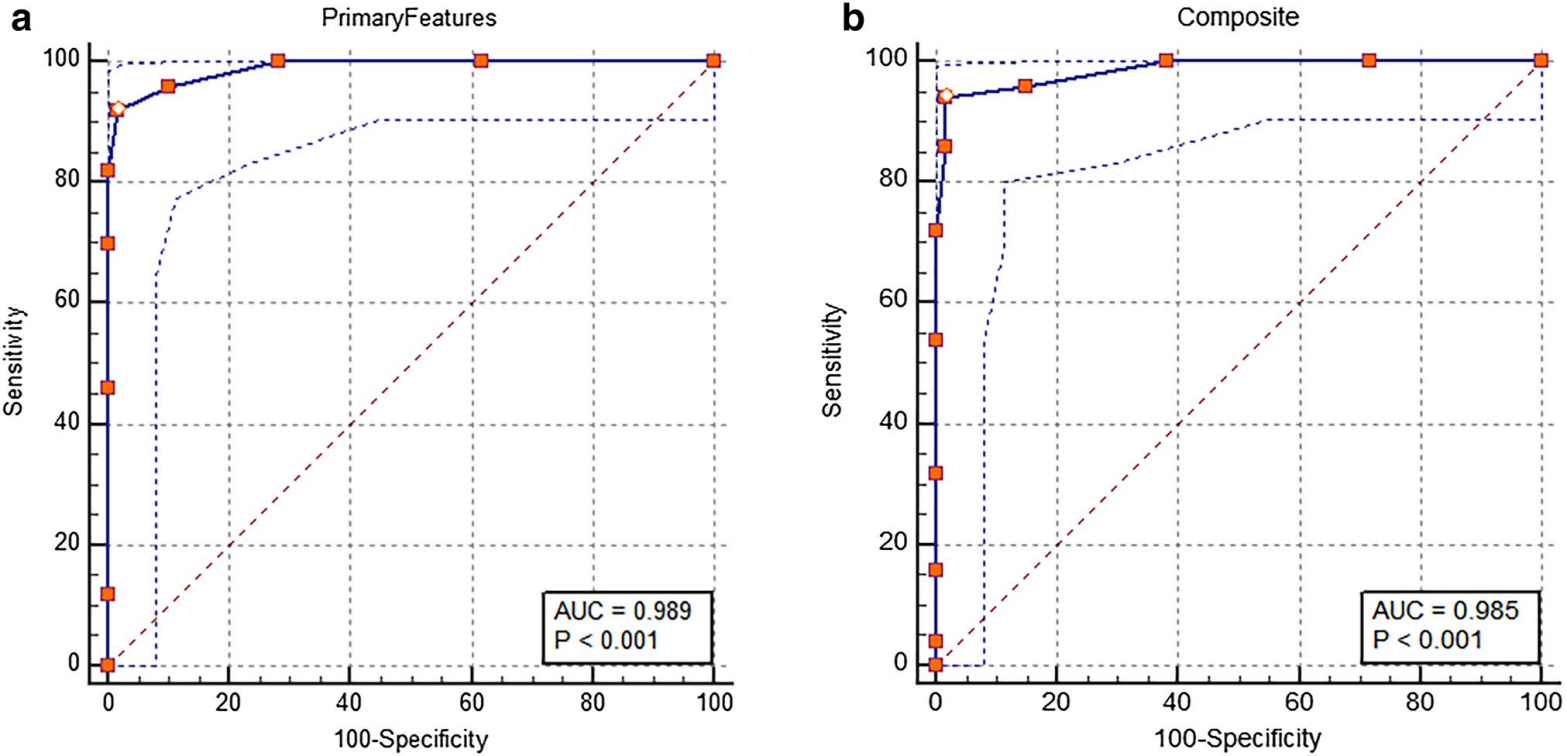
ROC curve analysis to predict SAA **a** Primary features with 95% CI, AUC (0.989) with sensitivity 92% and specificity 98%; **b** Composite severity score with 95% CI, AUC (0.985) with sensitivity 84–95% and specificity 91–98%

The binary logistic regression model analysis of FA revealed the following results: primary features OR = 7.7 (95% CI 2.8–21.0; *p* < 0.0001) for recognizing SAA; sensitivity of 92% and specificity of 98%. Secondary features and complications OR = 1.01 (95% CI 0.1–11.0; *p* = 0.99) as strong independent factors determining associated signs of progression; Composite severity score or SAA prediction score (SAAPS) OR = 6.2 (95% CI 2.7–14.0; *p* < 0.0001). However, 5–8 points (minimum-3 maximum-10) on SAAPS revealed a sensitivity of 84–95% and a specificity of 91–98% (Fig. 6a–b).

## Discussion

### A brief overview of the S. japonicum life cycle

The complete life cycle of schistosomal trematodes was first described by Pirajá da Silva in 1908 [21]. Oncomelania hupensis robertsoni, a subspecies of an aquatic gastropod mollusk, a very small freshwater snail serves as an intermediate host and a unique vector for *S. japonicum*, while humans serve as a definitive host in the transmission of Schistosomiasis. The eggs from feces and urine of infected individuals hatch and release miracidium into freshwater. They undergo developmental changes from sporocysts to cercaria. When humans come in contact with infested waters, cercaria loses the tail and become schistosomula that mature into sexually dimorphic adult worms. Adult worms mate in the hepatic parenchyma and move against the flow of blood to their final niche in the mesenteric circulation, where they begin egg production (> 32 days). Egg depositions occur intravascularly in the small venous tributaries of mesenteric and vesical plexus; eventually, they get migrated to the lumen of respective viscera aided by the peristaltic movement [22].

### Relation with intestinal schistosomiasis and age

*S. japonicum* eggs are the main pathogenic factor that gets deposited intravascularly in the portal venous system, as well as the small venous tributaries of the mesenteric or vesical plexuses. It has been well established that in the middle and late stages of the disease, the eggs often remain in clusters causing continuous erosion to the intestinal wall invading every layer of the rectum and sigmoid colon base forming long-segment circumferential interrupted mural calcifications [23]. Moreover, the presence of discontinuous to continuous calcifications extending along ascending colon and cecum could be attributed to the deposition of eggs from mesenteric plexus. Many of the patients that have been exposed to *S. japonicum* parasite, if not treated for a longer period of time, are liable to progress into SAA in later life years owing to gradual deposition of eggs in the appendicular sub-mucosal and sub-serosal layers.

In our study, some cases also showed one or more types of these calcifications on different MPR views suggesting single focus depositions are point calcifications, and surrounding sheet-like continuous and uniform hyper densities-linear calcifications, as well as annular hyper densities-orbital type calcifications occurring as a result of varying degrees of egg depositions at multiple foci on the surrounding layers of the appendix, out of which orbital calcifications are most common. In our study, it was also observed that the average age of the SAA patients who showed colon calcifications was 66.38 years. So, high age (> 53 years) with positive infection status for many years appears to be a major predisposing factor that could have contributed to a higher incidence of these vivid appendicular wall calcifications.

Petechial hemorrhages, granulomas in the lamina propria and submucosa and tram track/curvilinear calcifications are the most common lesions observed radiologically in intestinal schistosomiasis, similarly found in 90% cases of our study [24, 25]. However, secondary conditions such as Crohn’s disease, ulcerative colitis present with colon calcifications that are visualized as skip lesions, small bowel wall thickening, halos which are confined mostly to terminal ileum and cecum [26]. Intestinal tuberculosis shows circumferential wall thickening of terminal ileum and cecum, asymmetric thickening of the ileocecal valve along with mesenteric lymphadenopathy [27].

### Radiological analysis of the study

Plain CT is the preferred imaging modality for the detection of AA with high sensitivity (94–98%) and specificity up to 97% [28]. Dilated appendix with distended lumen (> 6 mm diameter), thickened and enhancing wall, thickening of the cecal apex (up to 80%): cecal bar sign, arrowhead sign, periappendiceal inflammation, including adjacent fat stranding and thickening of the lateroconal fascia or mesoappendix are mostly confirmatory features on NCCT and CCT [29]. Though the SAA group showed the highest positivity rate for these particular indicators, there were no significant differences. They are more common in young patients of either SAA or NSA because both groups manifested as the appendicular pathology. In our study, the SAA group showed appendiceal dilation > 13.6 mm and up to 22 mm along with various peculiar signs of complicated appendicitis suggesting ASA and AGA types are more frequent than simple appendicitis (SA).

### Pathophysiological correlation of radiological features

The role of schistosomiasis and the exact association in the development of appendicitis still remains uncertain and controversial [30] but we believe that it depends upon a series of changes triggered by two pathophysiological mechanisms after intestinal schistosomiasis infection that can be recognized as primary, secondary radiographic features, and its associated complications.

The first mechanism is the Schistosomal obstructive acute appendicitis-suppurative type. The basic structure of appendiceal orifice in the caecum and colon is similar, eggs deposited in the mesenteric plexuses eventually get migrated into the inferior mesenteric vein and superior hemorrhoidal vein leading to the tardy blood supply and decreased peristalsis of the appendix over time. At the same time, the peripheral intravenous eggs can get lodged into the appendicular lumen causing sluggish movements that can form appendicolith or fecalith and cause non-expulsion. Moreover, eggs can also get deposited in the appendiceal wall, submucosa, and muscular layer causing passive obstruction owing to fibrosis around eggs. Its primary structure is in the appendix wall but not on appendix lumen which is visualized as higher density peripheral to lumen—a point calcification resultant of such aggravated mechanism. It can also be further complicated by a bacterial infection with subsequent formation of a phlegmon or polyp, and sometimes with other conditions like intestinal tuberculosis, ileocecal intussusceptions, intestinal obstruction [31]. This mechanism pathologically presents with no tissue eosinophils or granulomas and is more often seen in the late stages of infection. It is always necessary to carefully evaluate for all the direct and indirect signs of acute appendicitis like effusions > 2.6 mm, fluid collections and peri appendiceal streak shadows (surrounding in-homogeneous appearance), inflammatory fat stranding, pneumatosis on CT [32].

The second mechanism is the acute Schistosomal appendicitis- granulomatous type, believed to be a result of immunological inflammatory and granulomatous reactions to newly deposited eggs [33] that are released in tandem along the submucosal layers of the appendix. Moreover, long-term deposition of eggs in the appendix wall is progressive increasing the granulomatous reaction and can form a kind of continuous homogeneous orbital calcification similar to that of the colon. When the underfilling of appendix lumen occurs, the calcification along the opposite sides of lumen shows as linear calcification because of partial volume effect and other restrictions. Even if the appendix collapses, it may appear as a line, but it is difficult to track the appendix when full. At the same time, 7 cases from the SAA group in which orbital calcifications has not been demonstrated, the degree of calcifications could directly be dependent on the quantity and duration of deposition of the eggs into the appendiceal cavity [34]. This mechanism presents pathologically with tissue necrosis, granulomas and eosinophilia, on the contrary, is more often seen in the early stages of infection.

The nidus for these granulomatous lesions is the parasite eggs with their multiple antigens that are recognized by the host. Peri appendiceal reactive nodal prominence, appendicular enlargement, and thickening, mucosal changes are precursors to perforation formation which can be recognized as a focal wall defect along with signs of suppurative type SAA [31]. Because such a vivid effect on the appendix is still uncommon in the endemic areas, it has been widely reported that the disease is progressing rapidly with higher perforation rates spontaneously, causing high mortality and morbidity [33].

Thus, we can infer that acute cases are at higher risk of perforation, granuloma, and abscess formation, while long-standing cases are most prone to the development of luminal fibrosis, appendicolith, and phlegmon formation. A simultaneous increase in age also precipitates the risk of progression into spontaneous perforations and peri-appendiceal abscess, the prognosis is poor [35]. A diameter greater than 13 mm appears to be a reliable feature in assessing appendicolith, fibrosis and abscess formation; while diameter greater than 18 mm can estimate the risk of eventual perforation in chronic cases. Calculating the radiological appendicitis severity index (APSI) [32] helps in differentiating SA, ASA, and AGA. There is a risk of misdiagnosis of SAA when the related signs are not analyzed in all anatomical planes.

Chronic infection can also result in scarring of mesenteric or vesicular blood vessels leading to portal hypertension, portocaval shunting [36] along with capsular calcifications extending towards the center of the liver with “turtleback appearance” due to massive fibrosis can also be observed in most cases [37] which are typical for hepatic schistosomiasis. There were no liver calcifications observed in the NSA group. Calcified eggs in the lymph nodes, the inflammatory tissues of mesocolon [15] and descending duodenum [38] appear as punctuate calcifications. There has been a well-established relation between Schistosomiasis and colorectal/rectosigmoid cancer [39]. It is still unknown that how many patients with hepatic or intestinal schistosomiasis are liable to develop into SAA, so, it is often very useful to look for these ancillary signs by exploring the adjacent abdominal viscera, especially liver and colon.

### Limitations of the study

SAA is a very rare entity that presents an acute emergency situation involving multiple complex pathophysiological mechanisms, so the sample size is slightly inadequate to analyze vivid patterns. Color doppler ultrasound is still the preliminary investigation of choice for diagnosing AA in a tertiary care level hospital. Contrast CT, fluoroscopy and MRI provides better diagnostic information; availability, technique, and expertise are major contributors for non-preference. Moreover, especially during, poor clinical conditions, perforations, and risk of side-effects to contrast materials are contraindications. We endeavor to integrate advanced modalities like Dual CT, MSCT with contrast studies, curved planar reformation (CPR) and CT virtual colonoscopy (CTVC), gastro intestinal fluoroscopy and selective angiography in our future projects aiming to completely understand the development and progression of SAA.

## Conclusion

Schistosomal associated appendicitis typically presents as suppurative and granulomatous types with a high incidence of peri appendiceal abscess formation and perforation especially in the elderly. Deposition of eggs in the sub-mucosal and sub-serosal layers of the appendix eventually gives rise to orbital, linear and point types of appendicular wall calcifications along with multiple colonic tram track calcifications. These radiographic features are typical for SAA and highly sensitive for early diagnosis, isolation. Features like larger appendicular diameter, pneumatosis, and focal wall defect are very useful for recognizing the type of reactions and the progression of the disease. Early recognition and management of SAA can prevent unanticipated complications and improve the prognosis of the patients.

## Abbreviations

AIR score: Appendicitis inflammatory response score
SAA: Schistosomal associated acute appendicitis
NSA: Non-schistosomal associated acute appendicitis
RLQBD: Right lower quadrant bowel dilatation
PCA: Principal component analysis
FA: Factor analysis
LR: Binomial logistic regression
MSCT: Multi slice computed tomography
MPR: Multi planar reconstruction
CPR: Curved planar reconstruction
